# Change Patterns During Family-Based Treatment for Pediatric Obsessive Compulsive Disorder

**DOI:** 10.1007/s10826-022-02479-8

**Published:** 2022-11-17

**Authors:** Chloe A. McGrath, Maree J. Abbott, Sharlene C. Mantz, Margot O’Brien, Daniel S. J. Costa, Felicity Waters

**Affiliations:** 1 School of Psychology, The University of Sydney, Sydney, NSW 2006 Australia; 2 Rivendell Child, Adolescent and Family Unit, Sydney Local Health District, Thomas Walker Estate, Hospital Road, Concord West, NSW 2138 Australia

**Keywords:** Obsessive compulsive disorder, Treatment, Cognitive behavior therapy, Child and adolescent, Family

## Abstract

Cognitive behavior therapy (CBT) for young people with obsessive compulsive disorder (OCD) has recently been enhanced to target family environment factors. However, the process of change for OCD symptoms and family factors during treatment is not well understood. Uniquely, we explored patterns of change for OCD symptoms and a range of family variables throughout Baseline, Early, Mid, and Late treatment phases of family-based CBT (FCBT) for 15 young people with OCD using multiple informants. We predicted a linear reduction in OCD symptom severity and family accommodation (FA) across treatment phases, however the investigation into other family factor change patterns was exploratory. OCD symptom severity, FA, parental distress tolerance (DT), and conflict all showed significant linear change patterns across treatment phases according to multiple informants. In addition, the largest proportion of change for these variables typically occurred during the first third of treatment, highlighting the importance of identifying participants with and without early gains in future research. Blame also showed a significant linear change pattern, although with small reductions between treatment phases. Preliminary bivariate analyses sought to better understand whether family factor change predicted subsequent OCD severity change or vice versa. Similar patterns emerged across informants, including identification of OCD severity as a significant predictor of change for Blame at subsequent treatment phases. Analyses also showed bi-directional effects for DT and OCD symptoms across informants, where DT predicted OCD severity at subsequent treatment phases and vice versa. These outcomes support further research aimed at understanding the role of family factors in pediatric OCD symptom change.

## Highlights

- This study explored patterns of change for OCD symptoms and family factors during family-based treatment for pediatric OCD.
- Findings provided converging support for linear change patterns for OCD symptoms and a range of family factors.
- Bivariate effects between family factor change and OCD symptom change during treatment were identified.
- Outcomes support further research investigating the role of family factors in pediatric OCD symptom change.

OCD in children and adolescents is a debilitating condition that can impair functioning across familial, social, academic, and occupational domains (American Psychiatric Association, 2013; Piacentini et al., 2003). Between 1 and 4% of young people are affected by the condition, which involves frequent obsessions and/or compulsions (Heyman et al., 2003; Storch et al., 2010; Valleni-Basile et al., 1995; Zohar, 1999). Obsessions are characterized by intrusive and recurring images, thoughts, or impulses that typically cause significant distress (American Psychiatric Association, 2013). Compulsions, or repetitive behaviors, are performed in an attempt to defuse the unwanted obsessions and related distress (American Psychiatric Association, 2013). OCD symptoms are time-consuming and can inhibit development that importantly occurs during childhood and adolescence (Piacentini et al., 2003; Storch et al., 2010).

Accumulating evidence suggests factors in the family environment affect the development and maintenance of OCD in young people. Family accommodation (FA), the involvement of a family member in an individual’s OCD symptoms, has been the most extensively researched family variable to date. FA can include modification of daily routines to accommodate OCD symptoms, assistance with the avoidance of anxiety-provoking situations, and active participation in symptoms (e.g., a parent handwashing more frequently to reduce child distress; Lebowitz et al., 2012). Providing reassurance and waiting for the completion of rituals are the most common forms of FA in child/adolescent OCD (Lebowitz et al., 2012). FA is negatively reinforced as child distress and the related parental distress are temporarily reduced. Although typically well-intentioned, FA maintains avoidance, anxiety, and OCD symptoms in the longer-term (Kagan et al., 2017, Lebowitz et al., 2014; Wu et al., 2016). FA rates are typically high in child/adolescent OCD, with most families engaging in frequent symptom accommodation. In a study by Flessner et al. (2011), 99% of families reported participating in at least one type of FA and 77.1% reported daily accommodation of symptoms. A significant correlation has been identified between the degree of FA and the severity of OCD symptoms (e.g., Lebowitz et al., 2012; Strauss et al., 2015; Wu et al., 2016). FA has also been linked to child functional impairment in a range of settings, including home, school, and social domains (e.g., Bipeta et al., 2013; Caporino et al., 2012; Thompson-Hollands et al., 2014).

Additional family environment factors have been associated with child/adolescent OCD, including criticism/blame of the young person, poor family problem-solving skills, and reduced distress tolerance (DT) in parents. Studies have shown high levels of criticism/blame are prevalent in OCD families (Hibbs et al., 1991; Mathieu et al., 2015; Peris et al., 2008; Przeworski et al., 2012). Blame of the young person has been correlated with OCD symptom severity, and particularly with the severity of OCD compulsions (Peris et al., 2008). Barrett et al. (2002) found parents with a child with OCD demonstrated less positive problem-solving compared to parents of a child with an anxiety disorder, an externalizing disorder, or no clinical diagnosis. Children with OCD showed the lowest levels of positive problem-solving during interactions with their parents compared to control groups. Although the research to date has been limited, DT in parents has been linked to child/adolescent anxiety and OCD. In a study by Selles et al. (2018), mothers and fathers were identified as having reduced tolerance of their OCD child’s distress pre-treatment (Selles et al., 2018). Similarly, Hudson et al. (2008) found parents with a child with an anxiety disorder were more reactive to their child’s expressions of distress (including anxiety and anger), demonstrating unhelpful responses such as criticizing, ignoring, disagreeing, becoming upset, and/or talking over the child, compared to parents of a child without an anxiety disorder. Lower levels of parental DT have also been correlated with higher levels of FA (Thompson-Hollands et al., 2014).

Family factors have also been linked to treatment response. Although cognitive behavior therapy (CBT) involving exposure and response prevention (ERP) has been well established as the first-line treatment for children and adolescents with OCD (Brauer et al., 2011; Freeman et al., 2018; Rosa-Alcázar et al., 2015), a large percentage of young people do not respond to the recommended treatment. The largest randomized controlled trial (RCT) to date examining treatment outcomes for children/adolescents with OCD highlighted a clinical remission rate of only 40% for the ERP-based CBT condition (POTS, 2004). Preliminary findings reveal family factors such as FA, DT, Conflict, and Criticism/Blame can affect the young person’s response to OCD treatment. In a large RCT, FA significantly predicted CBT outcomes for young people with OCD, such that families demonstrating lower levels of FA at baseline showed greater treatment response across conditions (including the ERP-based CBT condition; Garcia et al., 2010). Merlo et al. (2009) found change in FA pre- to post-CBT was significantly related to young people’s OCD severity at post-treatment, including after accounting for pre-treatment symptom severity. In a study by Gorenstein et al. (2015), young people with higher FA scores prior to treatment had poorer OCD symptom outcomes across CBT and fluoxetine treatment conditions. Francazio et al. (2016) found FA significantly predicted OCD symptom severity at both intake and 2-year follow-up and was the strongest predictor of severity at both time points. There is also data to suggest FA may even mediate OCD symptom outcomes (Piacentini et al., 2011). Piacentini et al. (2011) found changes in FA temporally preceded OCD symptom change in a RCT for children/adolescents with OCD. High maternal expressed emotion (EE), such as criticism and blame, at pre-treatment has been identified as a significant predictor of poor treatment response for young people with OCD (Peris et al., 2012b). Findings showed observed criticism of young people with OCD was also associated with increased OCD-related impairment post CBT (Przeworski et al., 2012). Similarly, Peris et al. (2012a) showed OCD-diagnosed young people from families with lower levels of blame and family conflict and higher levels of family cohesion prior to treatment were more likely to respond to CBT (93% response rate) compared to those with poorer functioning in all three domains (10% response rate). Parental DT may also affect treatment outcomes. In a recent study by Selles et al. (2018), fathers’ pre-treatment DT, and change in fathers’ DT over the course of treatment, were both significantly correlated with child OCD symptom improvement post-treatment.

In response to these emerging findings, interventions have recently been developed that directly target some of the family environment factors associated with OCD symptom improvement (e.g., Barrett et al., 2004; Peris & Piacentini, 2016; Piacentini et al., 2011). These enhanced family-based CBT (FCBT) interventions typically address FA. Additional family factors targeted by a few of these family-enhanced interventions include criticism/blame of the young person (e.g., Peris & Piacentini, 2016; Piacentini et al., 2011), problem-solving (e.g., Barrett et al., 2004; Peris & Piacentini, 2016), family conflict (Peris & Piacentini, 2016), and family cohesion (Peris & Piacentini, 2016). In studies where a number of family factors have been addressed, treatment outcomes have been excellent, evident from the large treatment effects reported (e.g., Barrett et al., 2004*d* = 2.65; Piacentini et al., 2011*d* = 2.37; Peris & Piacentini, 2013*d* = 2.59; Peris et al., 2017*d* = 2.07). A recent meta-analysis examining the effects of FCBT on treatment outcomes for child/adolescent OCD found the number of family factors actively targeted in treatment significantly moderated FA outcomes (McGrath & Abbott, 2019). Specifically, the greater the number of family factors addressed during treatment, the greater the reduction in the unhelpful family factor, FA, from pre- to post- treatment. This finding also has implications for OCD symptom outcomes as FA has been significantly correlated with OCD symptom severity (e.g., Lebowitz et al., 2012; Strauss et al., 2015; Wu et al., 2016). FCBT interventions have also shown improved outcomes compared to standard CBT with parental involvement. In a large RCT for young people with OCD, Peris and Piacentini (2016) reported a 68% treatment response rate and a 58% remission rate for the FCBT intervention compared to a 40% response rate and a 27% remission rate for standard CBT with parental involvement and psychoeducation.

Despite advances in OCD treatment interventions for young people, the process of symptom change during treatment is still not well understood. Typical pre-post assessment methodologies provide little information about the trajectory of change for OCD symptoms across treatment. As a result, little is known about how and when OCD severity changes during treatment and therefore the factors that could be implicated in these changes. Even less is known about the change processes of family factors and the associations between family factor change and OCD symptom change over the course of treatment. A better understanding of these change trajectories over time, and their associations, would assist in identifying key family factors to target in treatment, and those that could bolster OCD symptom change. Unfortunately, family factors are not assessed in the majority of treatment trials. Where measured, FA is typically assessed, and often only prior to treatment (McGrath & Abbott, 2019). Temporal tracking methodology, where variables are assessed at intervals throughout treatment, enables an examination of the trajectories of change for OCD symptoms and family factors over the course of treatment. Few studies in the child/adolescent OCD treatment literature have used this methodology to date, and mainly comprise case studies/series or assess distress during ERP tasks (e.g., Ginsburg et al., 2011; Kircanski et al., 2014; Knox et al., 1996). Piacentini et al. (2011) included a component of temporal tracking in a treatment trial comparing FCBT with relaxation training. Although not the primary focus of the study, preliminary findings showed changes in FA temporally preceded child-rated OCD symptom change. Further investigation is warranted to examine the patterns of change across treatment for OCD symptoms and a range of family factors, and to better understand temporal associations between family factors, including FA, and OCD symptom change. Change trajectories for additional family factors, such as DT, Blame, Conflict, and Cohesion, have not been examined. The current study contributes to the literature by addressing these identified gaps and limitations of previous studies.

## Aims and Objectives

The current study uses temporal tracking methodology to explore patterns of change for family factors and OCD symptoms across treatment phases during a well-regarded FCBT intervention for children and adolescents with OCD (Peris & Piacentini, 2013; Peris et al., 2017). This is the first pediatric OCD treatment trial to assess a range of family variables throughout Baseline, Early, Mid, and Late phases of treatment. The current study uniquely aims to identify patterns of change across treatment phases for the family factors, FA, DT, Blame, Conflict, and Cohesion. This study also uniquely explores associations between numerous family factors and OCD symptom severity across treatment phases, to better understand whether OCD symptoms change in relation to family factors or independently. In addition, the inclusion of multiple informants (i.e., child, parent, and clinician) further enhances the study’s strengths.

The study therefore aims to better understand: 1) univariate patterns of change across treatment phases for OCD symptoms and a range of family factors; 2) bivariate associations between family factor change and OCD symptom severity change across treatment phases. In relation to the first aim, we predicted a linear reduction in child-rated OCD symptom severity over the course of treatment, based on preliminary findings by Piacentini et al. (2011). We hypothesized that parent-rated and clinician-rated OCD symptom severity would similarly show linear reductions across treatment. Based on preliminary findings from a case series (Ginsburg et al., 2011), we hypothesized that FA would also reduce in a linear pattern across treatment phases. Other family factors are even less well understood, and the current investigation to better understand their change patterns across treatment was exploratory. In relation to the second aim, preliminary exploratory analyses using crossed-lagged panel modeling were employed to investigate the associations between family factor change and OCD symptom severity change across treatment phases, with a particular focus on whether family factor change preceded and predicted OCD symptom change. Based on preliminary findings by Piacentini et al. (2011), we hypothesized that a reduction in FA would predict OCD symptom reduction at the subsequent treatment phase. Based on prior literature (e.g., Peris et al., 2012a, b; Selles et al., 2018), we also hypothesized that a reduction in Blame would predict OCD symptom reduction and increases in DT would predict OCD symptom reduction. Hypotheses for the remaining family factors included an exploration of whether OCD symptoms changed independently of, or in relation to, family factors.

## Method

### Study Design and Inclusion Criteria

The current trial used temporal tracking methodology to measure changes in family variables and OCD symptom severity throughout Baseline, Early, Mid and Late treatment phases with multiple informants. Participants were monitored for a standard period of three weeks prior to commencing treatment and in this way served as their own controls. Young people and their parents completed a battery of questionnaires, assessing OCD symptom severity and family variables, weekly during the 3-week baseline period. The third baseline assessment occurred immediately prior to the first treatment session. Participants completed the same questionnaire battery, assessing the past week, prior to each of the remaining treatment sessions, and at post-treatment and 1-month follow-up. The study therefore employed a within-subjects repeated-measures design with 16 measurement timepoints. The 16 data points were consolidated into four treatment phases for the purpose of analyses (Baseline, Early Treatment, Mid Treatment, and Late Treatment) and follow-up. A well-established measure of OCD severity (National Institute of Mental Health Global Obsessive Compulsive Scale [NIMH-GOCS; Insel et al., 1983]) was completed by the treating clinician during each treatment phase throughout treatment. Young people between the ages of 8 and 17 years with a primary DSM-IV diagnosis of OCD were eligible to participate in the study (Diagnostic and Statistical Manual of Mental Disorders – Fourth Edition; American Psychiatric Association, 1994). Although they were assessed using the Anxiety Disorders Interview Schedule for DSM-IV – Child/Parent Versions (ADIS-IV-C/P; Silverman & Albano, 1996), participants also met DSM-5 diagnostic criteria for OCD (Diagnostic and Statistical Manual of Mental Disorders – Fifth Edition; American Psychiatric Association, 2013). Comorbid secondary diagnoses (e.g., anxiety disorders, depressive disorders) were permitted. Exclusion criteria included comorbid diagnoses of Tourette’s disorder, bipolar disorder, pervasive developmental disorders, psychosis, and other conditions contraindicated for the treatment intervention used and/or that could impair understanding of the treatment or measures, such as intellectual/cognitive impairments. Participants were required to be on a stable dose of psychotropic medication prior to commencing the trial and to maintain this stable dose throughout their involvement in the study. The study was approved by The University of Sydney (project no.:2014/462) and Sydney Local Health District (project no.:HREC/17/CRGH/116) research ethics review boards and registered with the Australia New Zealand Clinical Trials Registry (ANZCTR; Trial ID: ACTRN12614001272684).

### Participants

Participants were recruited via referrals from a pediatric OCD clinic and other pediatric community mental health organizations. Potential participants who were unable to adhere to the treatment schedule (e.g., regular attendance, parent involvement) were provided with alternative treatment options, such as referral to specialist practitioners at a university psychology clinic or to a pediatric OCD clinic providing both inpatient and outpatient treatment. The final sample comprised 15 young people (40% female) with a primary diagnosis of OCD (CSR ≥ 4). Participants’ ages ranged from 9 to 16 years, with a Mean of 14.1 years (SD = 2.0). The mean OCD clinician severity rating (CSR; ADIS-IV, Silverman & Albano, 1996) for participants prior to treatment was 5.9 (SD = 1.0) on a 0–8 scale (where 0 indicates no symptoms and 8 indicates severely disturbing/disabling symptoms). Approximately half of participants (47%, *n* = 7) had one or more comorbid diagnosis (*M* = 1.4 diagnoses, SD = 0.8), including generalized anxiety disorder (*n* = 4), social anxiety disorder (*n* = 3), separation anxiety disorder (*n* = 1), dysthymic disorder (*n* = 1), and attention deficit hyperactivity disorder (ADHD, *n* = 1). Most participants (73%) had received previous psychological treatment for OCD and/or other clinical disorders for a mean duration of 15.9 months (SD = 19.6). Half the participants (53%) were on one or more psychotropic medications and all maintained stable doses throughout treatment. In most cases, mothers (73%) were the primary caregiver involved in treatment. Mothers’ mean scores on a self-report measure of depression, anxiety, and stress symptoms (DASS-21) at pre-treatment fell within the moderate (*M* = 9.2, SD = 6.1), mild (*M* = 4.9, SD 5.4), and severe (*M* = 15.2, SD = 6.2) range, respectively. Father’s scores for the DASS-21 were calculated where available (47%): Mean scores fell within the moderate (*M* = 8.6, SD = 11.8), mild (*M* = 4, SD = 3.7), and moderate (*M* = 12.3, SD = 10) range for depression, anxiety, and stress symptoms, respectively.

### Measures

#### Pre-treatment, post-treatment, and follow-up measures

##### Diagnostic interview

Anxiety Disorders Interview Schedule for DSM-IV—Child/Parent Versions (ADIS-IV-C/P; Silverman & Albano, 1996): the ADIS-IV-C/P is a clinician-administered semi-structured interview used to diagnose anxiety and related disorders in young people according to DSM-IV diagnostic criteria. The ADIS-IV-C/P is commonly used in child anxiety research and has demonstrated excellent psychometric properties (Silverman et al., 2001). A clinician’s severity rating (CSR) of 4 or higher on a 0–8 scale denotes a clinically significant disorder. The ADIS-IV-C/P was used to identify clinical disorders, including OCD, in the present study and to evaluate severity at pre-treatment, post, and follow-up. Inter-rater reliability for primary diagnosis was calculated for 25% of participants randomly selected at pre- and at post- treatment using computer-generated random numbers. An independent evaluator (IE), a doctoral level clinical psychologist, blind to participants’ diagnostic status and to the condition of time (i.e., pre- versus post-treatment) reviewed child and parent video recordings of ADIS-IV interviews of the randomly-selected participant sample. Complete agreement (*κ* = 1.0) was obtained for primary diagnosis at both pre- and post-treatment time points, indicating excellent inter-rater reliability.

##### OCD symptoms

Children’s Yale-Brown Obsessive Compulsive Scale (CY-BOCS)—Child and Parent Report (PR): the CY-BOCS (Scahill et al., 1997) is a clinician-rated semi-structured interview evaluating OCD symptom severity in children and adolescents. The 10 items assess obsessions and compulsions using a 5-point scale. Total scores are categorized as signifying mild (8–15), moderate (16–23), severe (24–31), or extreme OCD symptoms (32–40) (Goodman et al., 1989). The CY-BOCS shows good reliability and validity (Scahill et al., 1997). Good internal consistency (*α* = 0.83) was calculated for the current sample. The CY-BOCS-PR (Storch et al., 2006) is a 10-item parent-report measure of OCD symptom severity based on the original CY-BOCS interview (Scahill et al., 1997*)*. The measure has adequate psychometric properties (Storch et al., 2006). Internal consistency for the current sample was fair (*α* = 0.78).

Child Obsessive Compulsive Impact Scale—Child/Parent Report (COIS-C/P; Piacentini et al., 2003): the child- and parent-report measures assess OCD-related impairment across various domains of functioning, including school, social, home/family domains. The degree of OCD interference is rated on a 4-point scale ranging from *not at all* to *very much*. The COIS demonstrates sound psychometric properties, including good construct validity and internal consistency (Piacentini et al., 2003). Internal consistency for the current sample was excellent (*α* = 0.93 child-report, *α* = 0.96 parent-report).

The National Institute of Mental Health Global Obsessive Compulsive Scale (NIMH-GOCS; Insel et al., 1983): the single item clinician-rated measure provides a global score of OCD symptom severity. The scale ranges from 1 (normal range) to 15 (very severe), where a rating of 7 or higher indicates clinical levels of OCD. The NIMH-GOCS has shown adequate to good convergent validity with the CY-BOCS and good to excellent test-retest reliability (Kim et al., 1993; Taylor, 1998).

*C*linical Global Impression—Improvement (CGI-I; Guy & Bonato, 1970): the CGI provides a rating of global improvement from baseline ranging from 1 (*very much improved*) to 7 (*very much worse*). Child, parent, and clinician ratings on the CGI-I were obtained post-treatment. Clinician ratings were obtained at follow-up. Ratings of 1 and 2 (*much improved*) were used to identify treatment responders, consistent with previous studies (e.g., Peris et al., 2017; Piacentini et al., 2011).

##### Family factors

Family Accommodation Scale—Parent Report (FAS-PR; Flessner et al., 2009): the 13-item parent-report measure assesses the degree to which family members accommodated OCD symptoms over the previous month using a 5-point scale, ranging from *never* to *daily*. The measure also assesses the associated level of parent and child impairment/distress. The FAS has shown good psychometric properties, including internal consistency (Calvocoressi et al., 1995, 1999). Internal consistency for the current sample was excellent (*α* = 0.92).

Parental Attitudes and Behaviors Scale (PABS; Peris et al., 2008): the PABS is a 24-item parent-report measure assessing family accommodation, blame, and empowerment. The three subscales are scored separately, with higher scores indicating higher levels of the respective family variables. The PABS has demonstrated good psychometric properties, including sound concurrent and predictive validity (Peris et al., 2008). The two subscales, Accommodation and Blame, were used in the current study. Internal consistency for the current sample was fair for Accommodation (*α* = 0.71) and good for Blame (*α* = 0.83).

Family Functioning Scales (FFS; Bloom, 1985): the 75-item FFS assesses family functioning across 15 dimensions (e.g., cohesion, conflict, expressiveness) using a 4-point scale ranging from *very untrue for my family* to *very true for my family*. The measure was developed using four well-known measures, including the Family Environment Scale (Moos & Moos, 1994). Each domain is scored separately, with higher scores indicating higher levels of the respective family variables. The FFS has sound psychometric properties (Bloom, 1985). The two domains, Cohesion and Conflict, were used in this study. Internal consistency for the current sample was good for child-rated Cohesion (*α* = 0.89) and Conflict (*α* = 0.82). However, internal consistency for parent-rated Cohesion (*α* = 0.50) and Conflict (*α* = 0.35) was poor and therefore these two parent-rated domains are not reported.

McMaster Family Assessment Device (FAD; Epstein et al., 1983): the 53-item FAD is a self-report measure assessing family functioning across six domains: problem-solving, communication, roles, affective responsiveness, affective involvement, and behavior control. Items are scored according to a 4-point rating scale, where higher scores indicate poorer levels of family functioning. The current study used the 12-item general functioning (GF) scale, which provides a summary of general family functioning, where a mean score below 2 indicates healthy family functioning. The FAD scales have sound reliability and have been shown to significantly distinguish between families that have been clinic-rated as “healthy” versus “unhealthy” (Epstein et al., 1983; Miller et al., 1985). Internal consistency for the current sample was excellent (*α* = 0.93 child-report; *α* = 0.92 parent-report).

##### Anxiety and depression symptoms

Children’s Depression Inventory (CDI; Kovacs, 1992): the 27-item child-report measure evaluates symptoms of depression over the previous two weeks using a 3-point scale; *t*-scores of 65 or higher are considered clinically significant. The measure has demonstrated good psychometrics, including internal consistency, test-retest reliability, and discriminative validity (Carlson & Cantwell, 1979). Internal consistency for the current sample was excellent (*α* = 0.90).

Depression Anxiety Stress Scale-21 (DASS-21; Lovibond & Lovibond, 1995): the 21-item self-report measure assesses the severity and frequency of depression, anxiety, and stress symptoms in adults over the previous week using a 4-point rating scale. Total scores for the depression, anxiety and stress scales fall between the ranges of *normal* and *extremely severe*. The measure has good reliability and validity (Antony et al., 1998). Internal consistency in the current sample was fair to good (*α* = 0.71 stress; *α* = 0.81 anxiety; *α* = 0.82 depression). Parents completed the measure at pre-treatment only.

#### Tracking Measures

##### OCD symptoms

OCD symptoms were assessed on a session-by-session basis throughout treatment using a modified version of the Children’s Obsessional Compulsive Inventory Revised (ChOCI-R; Shafran et al., 2003; Uher et al., 2008), which assesses the severity of obsessions and compulsions in young people. The ChOCI-R has sound psychometric properties, including internal consistency (Uher et al., 2008). The current study used the symptom scale, where scores range from 0 to 4, and an amended time frame of *the past week*. One obsession and one associated compulsion were tracked over the course of treatment for consistency purposes: The young person and their parent (with assistance from the assessing clinician as necessary) selected one of the primary obsessions and compulsions to rate. Total severity scores were obtained by adding the 6 items for obsessions and 6 items for compulsions, providing a maximum score of 48. Total scores of ≥12 (child-report) and ≥16–17 (parent-report) represent clinical levels of OCD. Internal consistency for the current sample ranged from fair to good (*α* = 0.77 child-report; *α* = 0.89 parent-report), in line with internal consistency results for the original ChOCI-R (child- and parent-report) symptom scale (e.g., Uher et al., 2008).

##### Family factors

The family tracking measure assessed multiple family variables over the previous week, including FA (parent-report), parental DT (parent-report), Blame of the young person (parent/child-report), Conflict (parent/child-report), and Cohesion (parent/child-report). The Parents’ Attitudes and Behaviors Scale (PABS; Peris et al., 2008) measured FA (parent-report) and Blame (parent-report). Subscales of the Family Functioning Scales (FFS; Bloom, 1985) were used for child and parent-rated Conflict and Cohesion. The assessment time frame of the PABS and FFS measures was modified to *the past week*. A one item measure was created to assess Blame (child-report), “My family blamed me for my OCD”, which was rated along a 4-point scale ranging from *very true* to *very untrue*. A one-item measure was created to assess parental tolerance of child distress (parent-report), “I found my child’s distress difficult to tolerate”. This item was rated on a 6-point scale, ranging from *not at all difficult to tolerate* to *completely difficult to tolerate*. Internal consistency for the modified PABS measure for the current sample was good for FA (*α* = 0.82) and Blame (*α* = 0.88 parent report), consistent with reliability findings reported by Peris et al. (2008). Internal consistency for the current sample for the modified FFS measure was good for Cohesion (*α* = 0.89 child report; *α* = 0.80 parent report) and acceptable for child-rated Conflict (*α* = 0.66), however poor for parent-rated Conflict (*α* = 0.55). No further analyses were therefore conducted for parent-rated Conflict.

### Treatment

Treatment was based on an FCBT intervention for young people with OCD, previously evaluated in two RCTs that both reported excellent treatment outcomes and maintenance of gains at 3-month follow-up (Peris & Piacentini, 2013; Peris et al., 2017). A recent systematic review and meta-analysis confirmed that this intervention directly targeted the largest number of family factors (McGrath & Abbott, 2019). Treatment included child (Piacentini et al., 2007) and family (Peris & Piacentini, 2016) components. The 12 one-hour child-focused sessions included ERP and cognitive restructuring. Family-focused sessions lasting an hour were held immediately after every alternate child session. Family sessions targeted specific family factors: FA, conflict, blame of the young person, problem-solving, and cohesion. At least one parent was required to attend all family-focused sessions together with the young person, although all immediate family members were invited to attend. On alternate weeks (where no family sessions were held) parents attended the last 10 to 15 min of the child session for a review. Treatment was provided by one of two registered clinical psychologists. Ten percent (*n* = 26) of treatment sessions were randomly selected using computer-generated random numbers and assessed for treatment fidelity. An IE, a doctoral level clinical psychologist experienced in the treatment of child and adolescent anxiety disorders, reviewed video recordings of treatment sessions to evaluate whether session content adhered to the treatment protocol. The IE was provided with a checklist of main content headings for each individual or family session identified from the detailed treatment manual (e.g., “Commence ERP with an item low on hierarchy”, “Normalize emotional responses to OCD”, “Introduce 3-step model for tough OCD episodes”). Each item was checked by the IE as present or absent in the session to ensure adherence to the manualized intervention. In addition, the IE indicated: a) whether or not the session fit within a CBT framework for pediatric OCD and b) whether or not the session included any non-CBT content as an additional assurance of fidelity. A treatment fidelity rating of 100% was obtained.

### Procedure

Referred families were invited to attend an appointment at one of two locations (i.e., a university psychology clinic, or a pediatric community mental health clinic) to complete informed consent and baseline assessments. After completing written informed consent, the young person and their parent/s were interviewed separately by clinical psychologists using the child/parent ADIS-IV and CY-BOCS (young person), to identify the presence and severity of clinical disorders, including OCD. Participants also completed a battery of self-report measures assessing OCD (and other clinical symptoms) and family variables. Eligible participants were assessed for three weeks prior to commencing treatment by completing weekly self-report tracking measures evaluating OCD symptoms and family factors over the previous week. Participants completed the third baseline assessment immediately prior to commencing the first session of the treatment intervention. Subsequent tracking measures were completed prior to the start of each remaining treatment session over the course of the 12-session intervention, as well as at post-treatment and at 1-month follow-up. Pre-treatment interviews and self-report measures were readministered at post-treatment. The tracking measures and a subset of the pre-post measures were sent to families at 1-month follow-up to complete at home and return via mail. Clinicians completed the NIMH-GOCS (Insel et al., 1983), a well-established clinician-rated measure of OCD symptom severity, at regular intervals through treatment, including at pre-treatment, after treatment sessions 4, 8, and 12 (post-treatment), and at follow-up. At post-treatment and follow-up timepoints the young person’s ADIS-IV and CY-BOCS interviews were re-administered by a non-treating registered psychologist/clinical psychologist in-person (post) or over the phone (follow-up). Data collection was conducted between May 2015 and July 2018. Treatment sessions were conducted weekly over 12-weeks, though, on occasion, sessions had to be rescheduled. Data collection was not impacted by the COVID-19 pandemic.

### Data Analysis

Repeated-measures ANOVA analyses were employed in SPSS (Version 26) to examine pre-post and post-follow-up treatment effects. Fifteen tracking data points, excluding follow-up (i.e., Timepoint 16), were consolidated for the purpose of data analysis into four data points corresponding with distinct treatment phases in order to maximize power, consistent with the majority of studies that reduce the number of data points when using equivalent analyses (e.g., Gregory et al., 2015). The three pre-treatment baseline monitoring datapoints were grouped together, and the 12 treatment datapoints were evenly grouped into thirds, in line with the structure of the treatment program which neatly fit into three distinct treatment phases (i.e., Early, Mid, and Late treatment). Treatment phases thus included: Phase 1: Baseline (Timepoint 1-Timepoint 3), Phase 2: Early Treatment (T4-T7), Phase 3: Mid Treatment (T8-T11), and Phase 4: Late Treatment (T12-T15). Mean scores were calculated for each of the four treatment phases for each variable assessed. Repeated-measures ANOVAs were used to identify univariate change trajectories for OCD symptom severity and for each family factor across the four treatment phases. Pearson’s *r* correlations, assessing bivariate associations between each family variable and OCD symptom severity, were calculated for each of the four treatment phases. In addition, Pearson’s *r* correlations were used to investigate the association between each family factor at a specific treatment phase and OCD symptom severity at the subsequent phase. Crossed-lagged panel analyses were used to explore reciprocal associations between family factor change and OCD symptom severity change over the course of treatment, whilst controlling for the stability of each variable over time. The model examines whether one variable predicts change in another variable, and vice versa. For example, cross-lagged regression coefficients indicate how much variance in Variable *a* at Time 1 predicts change in Variable *b* between Time 1 and Time 2. The inverse is also calculated, namely how much variance in Variable *b* at Time 1 predicts change in Variable *a* between Time 1 and Time 2. In particular, the analyses examined whether a family factor at a nominated treatment phase was associated with corresponding changes in OCD symptom severity at the subsequent treatment phase. The model also inversely examined whether OCD symptom severity predicted changes in a family factor at the subsequent treatment phase. A four-wave crossed-lagged analysis was conducted using the four distinct treatment phases: Time lag one: Baseline, time lag two: Early Treatment, time-lag three: Mid Treatment, and time-lag four: Late Treatment. Crossed-lagged panel analyses were conducted in MPlus Version 7 (Muthén & Muthén, 1998–2012). Maximum likelihood (ML) estimation, the fitting function most commonly used for structural equation models, was used in the current study. Maximum likelihood (ML) estimation provides parameter estimates, along with standard errors and a mean-adjusted chi-square test statistic, that are robust to non-normality. Model fit was evaluated using the comparative fit index (CFI; Bentler, 1990). The CFI statistic has been identified as one of the fit indices least affected by sample size (Schermelleh-Engel et al., 2003), and is a better indicator of model fit compared to the RMSEA index when sample sizes are smaller and analyses are exploratory rather than confirmatory (Rigdon, 1996). The CFI tends to avoid the underestimation of fit often found with small samples (Schermelleh-Engel et al., 2003). The Chi-squared statistic is very sensitive to sample size and is no longer recommended as a basis for acceptance or rejection of the null hypothesis/model fit (Schermelleh-Engel et al., 2003; Vandenberg, 2006). CFI values of 0.90 or larger denoted satisfactory model fit (Rigdon, 1996).

#### Missing Data

Missing data for weekly tracking measures were less than 1.7 % in total across the 16 data points. The few missing items were replaced with the mean of the respective preceding and succeeding data points. Missing data for measures given at pre, post, and follow-up were minimal. At post, one parent missed the CY-BOCS-PR and one child missed the FAD (0.8%). At follow-up, one participant failed to complete the designated measures and one parent did not complete the COIS-P (7.3%). Where participants completed 80% or more of questionnaire items, missing items were replaced with mean scores.

## Results

### Pre-, Post-Treatment, and Follow-up Outcomes

The results of repeated-measures ANOVAs for symptom and family factor measures are reported in Table 1, including pre-post and post-follow-up comparisons. Table 1 reports pre-treatment, post-treatment, and follow-up means and standard deviations, *F*-test values and significance levels, and Cohen’s *d* effect sizes. All symptom measures (i.e., CY-BOCS, CY-BOCS-PR, ADIS-IV CSR, NIMH-GOCS, COIS-C/P, CDI) showed significant reductions in scores pre- to post- treatment. All measures evaluating the severity of OCD symptoms (i.e., CY-BOCS, CY-BOCS-PR, ADIS-IV CSR, NIMH-GOCS) demonstrated very large Cohen’s *d* effect sizes, ranging from *d* = 1.30 to *d* = 1.82. Medium to large effect sizes were found for OCD measures assessing OCD interference (*d* = 0.55 COIS-C, *d* = 0.77 COIS-P). Family factor measures that showed significant pre-post outcomes included: FAS, PABS-Accommodation, PABS-Blame, and FFS-Conflict (child-report). Medium to large effect sizes were calculated for these family factors, ranging from *d* = 0.65 (FFS-Conflict-C) to *d* = 1.18 (PABS-Accommodation). Family measures that did not demonstrate significant post-treatment change included: FAD-GF-C/P and FFS-Cohesion-C. General family functioning (FAD-GF) showed small improvements pre- to post- treatment (*d* = 0.34 child-report, *d* = 0.11 parent-report), however FFS-Cohesion-C did not improve pre-post (*d* = 0.00). All treatment gains were maintained at one-month follow-up. Additional significant treatment gains were evident on the NIMH-GOCS (*d* = 0.40) and COIS-C (*d* = 0.54) at follow-up.

**Table 1:** Pre-post and post-follow-up comparisons

	Pre-post				Post-follow-up		
	Pre *M* (SD)	Post *M* (SD)	*F*	*d*	F/up *M* (SD)	*F*	*d*
Symptom measures							
CY-BOCS	22.9 (6.3)	13.6 (7.9)	37.97***	1.30	11.2 (8.0)	2.24	0.28
CY-BOCS-PR	26.2 (4.8)	14.9 (8.9)	48.09***	1.58	13.9 (8.2)	0.06	0.04
ADIS-IV CSR (OCD)	5.9 (1.0)	3.3 (1.7)	36.96***	1.82	2.6 (1.7)	4.22	0.27
NIMH-GOCS	8.8 (1.4)	5.0 (2.6)	53.77***	1.82	3.6 (2.6)	6.84*	0.40
COIS-C	28.9 (20.0)	15.3 (15.0)	7.96**	0.77	9.6 (9.9)	7.03*	0.54
COIS-P	37.1 (21.1)	24.7 (23.9)	8.22**	0.55	17.3 (15.0)	0.01	0.02
CDI	4.5 (4.2)	2.7 (3.4)	5.84*	0.48	–	–	–
Family measures							
FAS	24.0 (12.8)	14.7 (12.0)	14.28**	0.75	11.4 (8.9)	0.69	0.16
PABS-A	20.2 (5.9)	13.5 (5.5)	22.70***	1.18	–	–	–
PABS-B	17.0 (5.7)	13.1 (4.1)	10.14**	0.78	–	–	–
FAD-GF-C	1.9 (0.7)	1.6 (0.7)	1.93	0.34	1.5 (0.5)	2.17	0.24
FAD-GF-P	2.1 (0.5)	2.0 (0.8)	0.32	0.11	1.9 (0.5)	0.32	0.10
FFS-Conflict-C	11 (3.9)	8.7 (3.1)	6.06*	0.65	–	–	–
FFS-Cohesion-C	16.7 (3.2)	16.7 (3.4)	0.00	0.00	–	–	–

*M* mean*, SD* standard deviation*, F* *F*-test, *d* Cohen’s *d*, *F/up* follow-up, *-C* child, *-P* parent, *CY-BOCS* Children’s Yale-Brown Obsessive Compulsive Scale, *-PR* parent-report, *ADIS CSR* Anxiety Disorders Interview Schedule Clinician’s Severity Rating, *NIMH-GOCS* The National Institute of Mental Health Global Obsessive Compulsive Scale, *COIS* Child Obsessive-Compulsive Impact Scale, *CDI* Children’s Depression Inventory, *FAS* Family Accommodation Scale, *PABS-A/B* Parental Attitudes and Behaviors Scale-Accommodation/Blame, *FAD-GF* McMaster Family Assessment Device-General Functioning scale, *FFS* Family Functioning Scales

*p ≤ 0.05; **p ≤ 0.01; ***p ≤ 0.001

### Treatment Response and Remission

Eighty percent of participants (12/15) were identified as treatment responders on the CGI-I at post-treatment. Participants were categorized as responders when at least two of the three raters (i.e., parent, child, clinician) provided a score of 1 (very much improved) or 2 (much improved) on the CGI-I. Clinician-rated CGI-I scores examined independently yielded the same results as above, classifying 80% of participants (12/15) as treatment responders. At follow-up, 86% of participants (12/14) were identified as treatment responders on the clinician-rated CGI-I. At post-treatment, 67% of participants (10/15) achieved remission, no longer meeting diagnostic criteria for OCD on the ADIS-IV-C/P (CSR < 4). Similarly, 67% of participants (10/15) obtained a score below the clinical range (<7) for OCD on the NIMH-GOCS. At follow-up, 71% of participants (10/14) were identified at remitters based on the ADIS-IV-C. Similarly, 71% of participants (10/14) scored below the clinical range for OCD on the NIMH-GOCS.

### Univariate Change Trajectories Across Treatment

**Fig. 1:**
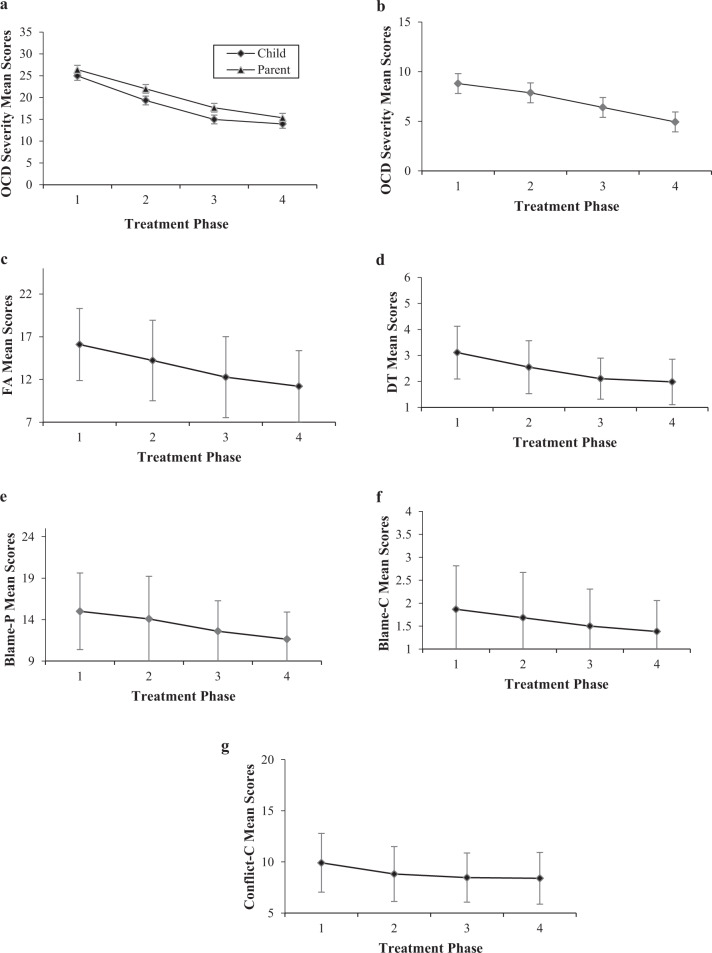
**a** Mean child- and parent-rated OCD severity scores and standard deviations at each treatment phase. **b** Mean clinician-rated OCD severity scores and standard deviations at each treatment phase. **c** Mean parent-rated family accommodation (FA) scores and standard deviations at each treatment phase. **d** Mean parent-rated distress tolerance (DT) scores and standard deviations at each treatment phase. **e** Mean parent-rated blame scores and standard deviations at each treatment phase. **f** Mean child-rated blame scores and standard deviations at each treatment phase. **g** Mean child-rated conflict scores and standard deviations at each treatment phase

Means and standard deviations for OCD symptom severity (child/parent/clinician report) across the four phases of treatment (i.e., Baseline, Early Treatment, Mid Treatment, Late Treatment) are depicted in Fig. 1a, b. Figure 1c–g shows the family variables that demonstrated significant change trajectories across treatment, namely FA (parent-report), DT (parent-report), Blame (child- and parent- report), and Conflict (child-report). Repeated-measures ANOVA results demonstrated significant linear reductions in mean scores across treatment for OCD symptom severity, according to multiple informants: Child (*F* (1,14) = 17.91, *p* < 0.001), parent (*F* (1,14) = 72.68, *p* < 0.001), and clinician (*F* (1,14) = 57.93, *p* < 0.001). Similarly, FA (*F* (1,14) = 52.77, *p* < 0.001) and DT (i.e., the degree of difficulty parents experienced tolerating their child’s distress); *F* (1,14) = 22.43, *p* < 0.001) both showed significant linear reduction in mean scores over the course of treatment. Significant linear patterns of change were also evident for child-rated Blame (*F* (1,14) = 7.66, *p* = 0.02) and parent-rated Blame (*F* (1,14) = 12.02, *p* = 0.004). Child-rated Conflict data fitted both a significant linear (*F* (1,14) = 6.84, *p* = 0.02) and a significant quadratic (*F* (1,14) = 4.72, *p* = 0.048) pattern of change across treatment. Conflict scores reduced linearly between Baseline and Mid Treatment and then levelled off between Mid and Late Treatment, also consistent with a quadratic component. Child- and parent- rated Cohesion showed non-significant change trajectories across treatment (*p* > 0.05).

Table 2 reports Cohen’s *d* effect size calculations, showing the degree of change that occurred between each of the four treatment phases, for OCD symptom severity and for family factors with significant change trajectories (i.e., FA, DT, Blame, and Conflict). Clinician-rated OCD symptom severity showed medium to large effect sizes between treatment phases, with the largest reduction in OCD severity evident between Early and Mid Treatment. Child- and parent- rated OCD severity showed similar patterns of change: the largest reductions in severity occurred between the first three treatment phases (i.e., between Baseline and Mid Treatment), where medium effect sizes were generally evident, and the smallest amount of change occurring between Mid and Late Treatment, where small effect sizes were calculated. Similarly, the family factors, FA and DT, showed greater change between the first three treatment phases (where small to medium effect sizes were calculated) compared to between Mid and Late Treatment (where small effects sizes were calculated). Effect size calculations for child-rated Conflict also showed the majority of changed occurred between the first three treatment phases (particularly between Baseline and Early Treatment). The reduction in Conflict then levelled off between Mid and Late Treatment, where almost no change occurred. Child- and parent- rated Blame showed small effect sizes between all four treatment phases.

**Table 2:** Cohen’s *d* effect size calculations between subsequent treatment phases

	Phase 1–2 (*d*)	Phase 2–3 (*d*)	Phase 3–4 (*d*)
OCD symptoms			
OCD—child	0.58	0.48	0.11
OCD—parent	0.46	0.43	0.20
OCD—clinician	0.62	0.80	0.64
Family factors			
FA	0.42	0.41	0.24
DT	0.55	0.49	0.15
Blame-child	0.19	0.20	0.20
Blame-parent	0.19	0.33	0.28
Conflict-child	0.39	0.14	0.03

*d* Cohen’s *d*, *OCD* obsessive compulsive disorder, *FA* family accommodation, *DT* distress tolerance

### Exploratory Analyses of Bivariate Associations

Refer to Table 3a,b for bivariate correlations. In general, family factors were mildly to strongly positively correlated with OCD severity at each of the four treatment phases (Table 3a) and with OCD severity at the subsequent treatment phase (Table 3b). Overall, FA and DT were the family factors with the strongest correlations with OCD symptom severity, followed by child-rated Blame.

**Table 3:** Correlations between family factors and OCD symptoms at each treatment phase (a) and at subsequent treatment phases (b)

a. Correlations between family factors and OCD symptoms at each treatment phase					
FF × parent-rated OCD	*r*	FF × child-rated OCD	*r*	FF × clinician-rated OCD	*r*
FA 1 × pOCD 1	0.58*	FA 1 × chOCD 1	0.06	FA 1 × cOCD 1	0.38
FA 2 × pOCD 2	0.78**	FA 2 × chOCD 2	0.35	FA 2 × cOCD 2	0.58*
FA 3 × pOCD 3	0.71**	FA 3 × chOCD 3	0.30	FA 3 × cOCD 3	0.45
FA 4 × pOCD 4	0.64*	FA 4 × chOCD 4	0.06	FA 4 × cOCD 4	0.37
DT 1 × pOCD 1	0.46	DT 1 × chOCD 1	0.48	DT 1 × cOCD 1	0.50
DT 2 × pOCD 2	0.69**	DT 2 × chOCD 2	0.61*	DT 2 × cOCD 2	0.70**
DT 3 × pOCD 3	0.82**	DT 3 × chOCD 3	0.56*	DT 3 × cOCD 3	0.59*
DT 4 × pOCD 4	0.85**	DT 4 × chOCD 4	0.21	DT 4 × cOCD 4	0.49
chBlame 1 × pOCD 1	0.44	chBlame 1 × chOCD 1	0.75**	chBlame 1 × cOCD 1	0.31
chBlame 2 × pOCD 2	0.66**	chBlame 2 × chOCD 2	0.62*	chBlame 2 × cOCD 2	0.64*
chBlame 3 × pOCD 3	0.59*	chBlame 3 × chOCD 3	0.49	chBlame 3 × cOCD 3	0.5
chBlame 4 × pOCD 4	0.48	chBlame 4 × chOCD 4	0.52*	chBlame 4 × cOCD 4	0.62*
pBlame 1 × pOCD 1	0.37	pBlame 1 × chOCD 1	0.05	pBlame 1 × cOCD 1	0.19
pBlame 2 × pOCD 2	0.59*	pBlame 2 × chOCD 2	0.36	pBlame 2 × cOCD 2	0.51
pBlame 3 × pOCD 3	0.44	pBlame 3 × chOCD 3	0.18	pBlame 3 × cOCD 3	0.33
pBlame 4 × pOCD 4	0.36	pBlame 4 × chOCD 4	−0.15	pBlame 4 × cOCD 4	0.13
cConflict 1 × pOCD 1	0.56*	cConflict 1 × chOCD 1	0.60*	cConflict 1 × cOCD 1	0.66**
cConflict 2 × pOCD 2	0.46	cConflict 2 × chOCD 2	0.16	cConflict 2 × cOCD 2	0.48
cConflict 3 × pOCD 3	0.31	cConflict 3 × chOCD 3	−0.00	cConflict 3 × cOCD 3	0.16
cConflict 4 × pOCD 4	0.42	cConflict 4 × chOCD 4	−0.08	cConflict 4 × cOCD 4	0.24

b. Correlations between family factors and OCD symptoms at the subsequent treatment phase					
FF × parent-rated OCD	*r*	FF × child-rated OCD	*r*	FF × clinician-rated OCD	*r*
FA 1 × pOCD 2	0.55*	FA 1 × chOCD 2	0.32	FA 1 × cOCD 2	0.38
FA 2 × pOCD 3	0.73**	FA 2 × chOCD 3	0.26	FA 2 × cOCD 3	0.57*
FA 3 × pOCD 4	0.61*	FA 3 × chOCD 4	0.17	FA 3 × cOCD 4	0.45
DT 1 × pOCD 2	0.42	DT 1 × chOCD 2	0.68**	DT 1 × cOCD 2	0.57*
DT 2 × pOCD 3	0.57*	DT 2 × chOCD 3	0.52*	DT 2 × cOCD 3	0.66**
DT 3 × pOCD 4	0.71**	DT 3 × chOCD 4	0.36	DT 3 × cOCD 4	0.56*
chBlame 1 × pOCD 2	0.43	chBlame 1 × chOCD 2	0.75**	chBlame 1 × cOCD 2	0.46
chBlame 2 × pOCD 3	0.66**	chBlame 2 × chOCD 3	0.51	chBlame 2 × cOCD 3	0.51
chBlame 3 × pOCD 4	0.58*	chBlame 3 × chOCD 4	0.33	chBlame 3 × cOCD 4	0.42
pBlame 1 × pOCD 2	0.61*	pBlame 1 × chOCD 2	0.40	pBlame 1 × cOCD 2	0.41
pBlame 2 × pOCD 3	0.46	pBlame 2 × chOCD 3	0.45	pBlame 2 × cOCD 3	0.61*
pBlame 3 × pOCD 4	0.28	pBlame 3 × chOCD 4	0.07	pBlame 3 × cOCD 4	0.34
cConflict 1 × pOCD 2	0.53*	cConflict 1 × chOCD 2	0.43	cConflict 1 × cOCD 2	0.51
cConflict 2 × pOCD 3	0.60*	cConflict 2 × chOCD 3	0.07	cConflict 2 × cOCD 3	0.38
cConflict 3 × pOCD 4	0.34	cConflict 3 × chOCD 4	−0.13	cConflict 3 × cOCD 4	0.20

*FF* family factor, *r* Pearson’s *r*, *FA* family accommodation, *1* phase 1, *2* phase 2, *3* phase 3, *4* phase 4, *p* parent, *ch* child, *c* clinician, *DT* distress tolerance

*p ≤ 0.05; **p ≤ 0.01

Preliminary analyses, using crossed-lagged panel modeling, were used to explore reciprocal associations between family factors and OCD severity over the course of treatment. The bivariate associations described below included those where the majority of the models showed good fit of the data, namely FA, DT, and child-rated Blame. These family factors also tended to show stronger correlations with OCD severity (Table 3a, b). Model results (unstandardized and standardized) for the significant predictors of change identified are presented in Table 4.

#### FA × OCD Severity

The models, FA and child-rated OCD severity (RMSEA = 0.19, CFI = 0.95) and FA and clinician-rated OCD severity (RMSEA = 0.22, CFI = 0.95), both had good fit with the respective data, however no significant predictors of change were identified for the current sample. The model for FA and parent-rated OCD severity did not show adequate fit with the current data (RMSEA = 0.50, CFI = 0.77).

#### DT × OCD Severity

Models for DT and OCD symptom severity had good fit with the data across all three informants. The model for DT and parent-rated OCD severity (RMSEA = 0.24, CFI = 0.94) identified OCD severity at Baseline, Early Treatment, and Mid Treatment as significant predictors of DT at subsequent treatment phases (i.e., Early, Mid, and Late Treatment, respectively). In addition, the same model identified DT at Early Treatment as a significant predictor of change for OCD severity at the subsequent treatment phase, Mid Treatment. The model for DT and clinician-rated OCD severity (RMSEA = 0.23, CFI = 0.93) also highlighted OCD severity at Baseline as a significant predictor of change for DT at Early Treatment. The model for DT and child-rated OCD severity (RMSEA = 0.24, CFI = 0.92) identified DT at Baseline as a significant predictor of change for OCD severity at Early Treatment.

#### Child-rated Blame × OCD Severity

The model for Blame and parent-rated OCD severity had good fit with the data (RMSEA = 0.12, CFI = 0.98). The model highlighted OCD severity at Baseline as a significant predictor of change for Blame at the subsequent treatment phase, Early Treatment. Child-rated OCD severity (RMSEA = 0.30, CFI = 0.89) and clinician-rated OCD severity (RMSEA = 0.32, CFI = 0.89) models both approached good fit of the data. The clinician-rated OCD severity model similarly identified OCD severity at Baseline as a significant predictor of change for Blame at Early Treatment. The child-rated OCD severity model identified OCD severity at Mid Treatment as a significant predictor of Blame at Late Treatment.

**Table 4:** Predictors of change across treatment phases showing significant results for unstandardized and standardized models

		Estimate		SE	
Model	Predictors	*b*	*β*	*b*	*β*
DT × pOCD	OCD 1 -> DT 2	0.03*	0.3	0.02	0.19
DT × pOCD	OCD 2 -> DT 3	0.06***	0.69***	0.02	0.17
DT × pOCD	OCD 3 -> DT 4	0.05*	0.53*	0.02	0.24
DT × pOCD	DT 2 -> OCD 3	−1.66***	−0.16*	0.39	0.07
DT × cOCD	OCD 1 -> DT 2	0.23**	0.32**	0.09	0.12
DT × chOCD	DT 1 -> OCD 2	3.22**	0.36*	1.14	0.14
chBlame × pOCD	OCD 1 -> chB 2	0.03	0.28*	0.02	0.14
chBlame × cOCD	OCD 1 -> chB 2	0.18*	0.26	0.09	0.16
chBlame × chOCD	OCD 3 -> chB 4	0.02*	0.31*	0.01	0.14

*SE* standard error, *b* unstandardized, *β* standardized, *DT* distress tolerance, *OCD* obsessive compulsive disorder, *p* parent, *c* clinician, *ch* child, *B* blame, -> predicted, *1* phase 1, *2* phase 2, *3* phase 3, *4* phase 4, *OCD 1* *->* *DT 2* OCD phase 1 predicted DT at phase 2

*p ≤ 0.05; **p ≤ 0.01; ***p ≤ 0.001

## Discussion

The current study used temporal tracking methodology to explore family factor change and OCD symptom severity change throughout Baseline, Early, Mid, and Late treatment phases of an FCBT intervention for young people with OCD. The preliminary study uniquely aimed to better understand univariate patterns of change across treatment for a range of family factors and OCD symptoms using multiple informants. The study also uniquely explored bivariate associations between family factor change and OCD symptom severity change across treatment phases using a range of family factors and aimed to better understand whether OCD symptoms changed in relation to family factors or independently.

The treatment response rate for the current trial was 80% at post-treatment and 86% at follow-up. The remission rate was 67% at post and 71% at follow-up. This is the first independent replication of the FCBT intervention used by Peris and Piacentini (2013) and Peris et al. (2017), with the current trial showing comparable outcomes. OCD symptoms reduced significantly pre- to post- treatment on all OCD measures according to child, parent, and clinician report, and, in particular, showed very large effect sizes for OCD severity change. Family factors, including FA, Blame, and child-rated Conflict, showed significant changes pre- to post- treatment, with the exception of Cohesion and General Family Functioning.

OCD symptom severity and the family factors, FA, DT, and Conflict all showed significant linear patterns of change across treatment phases according to multiple informants. Effect size calculations for these variables (parent- and child- report) showed the majority of change occurred during the first two thirds of treatment (i.e., between Baseline and Mid Treatment), encompassing the first 8 sessions of treatment. Furthermore, the greatest amount of change for these variables typically occurred during the first third of treatment (i.e., between Baseline and Early treatment), comprising the first four treatment sessions. These results are in line with findings that highlight the importance of early treatment response for positive treatment outcomes (e.g., Beard & Delgadillo, 2019). Early response is commonly defined as symptom change during the first four sessions/first month, although some studies refer to change during the initial eight sessions (Beard & Delgadillo, 2019). The current results also extend previous findings, which typically focus on anxiety and depression symptoms in adults, by highlighting the importance of early response for young people regarding both OCD symptoms and family factors. Furthermore, these findings indicate the importance of identifying characteristics distinguishing participants who show early treatment gains, as well as those who fail to show substantial gains in the early phase of treatment, in future research. The family factor, Blame, also showed a significant linear pattern of change across treatment, although small reductions between each of the four treatment phases were evident, with slightly less change apparent during the first third of treatment according to parent report. A possible explanation for the initial lag in parent-reported blame reduction is that OCD symptom change precedes and predicts change in Blame, supported by preliminary findings from the exploratory crossed-lagged panel analyses. Once the child starts participating in treatment and shows initial improvements in OCD symptoms, it may be that parents feel less blame towards their child for these symptoms. Alternatively, the first few sessions involve psychoeducation for the family, including about factors that can contribute to the development and maintenance of OCD (e.g., the OCD cycle, stressful events, the hereditary nature of anxiety). Parents’ enhanced understanding of the range of factors that can influence OCD development and maintenance, gained during the first third of treatment, could subsequently result in the child being less the focus of blame for their OCD symptoms. The findings for FA, DT, Conflict, and Blame extend previous findings by identifying change trajectories for a range of family factors and highlighting the specific periods during treatment where the majority of change occurred for these variables. Cohesion was the only family factor that showed non-significant change patterns across treatment, although it is worth noting that high levels of Cohesion were already present in the current sample at pre-treatment.

Exploratory cross-lagged panel analyses highlighted significant bivariate associations between family factor change and OCD symptom change during treatment and similar patterns across multiple informants for respective variables. Models exploring associations between DT and OCD symptom severity identified several predictors of change. Reduced OCD severity at Baseline, Early Treatment, and Mid Treatment significantly predicted a reduction in parents’ difficulty tolerating their child’s distress at each subsequent treatment phase (i.e., Early, Mid, and Late Treatment). Consistent with our expectations that an increase in DT would predict OCD symptom reduction, and furthermore, in support of bi-directional conceptualizations, the child-rated OCD severity model also identified DT at Baseline as a significant predictor of subsequent change for OCD severity. Greater tolerance of child distress at Baseline predicted a reduction in OCD severity at the subsequent treatment phase, Early Treatment. Parent-rated OCD severity also identified DT at Early Treatment as a significant predictor of change for OCD severity at the subsequent treatment phase, Mid Treatment, whereby parents’ increased difficulty tolerating their child’s distress in the Early Treatment phase predicted a decrease in OCD severity at the subsequent treatment phase. This pattern corresponds with the introduction of ERP in Early Treatment and may explain parents’ increased difficulty tolerating their child’s distress, which may be linked to the likely increase in child distress as the young person commences in-session and home-based ERP tasks (involving exposure to feared stimuli) for the first time. Therefore, it may be that young people’s participation in these early ERP tasks (rather than avoidance of these tasks and the associated distress) is associated with greater parental difficulty tolerating increased child distress, but that engaging in ERP nonetheless predicts reduced OCD severity at the next treatment phase. The aforementioned DT findings, which showed that parent’s DT at Baseline and at Early Treatment predicted OCD severity at the subsequent treatment phases (i.e., Early Treatment and Mid Treatment), suggest a bivariate association for these variables in line with findings by Selles et al (2018), which showed that father’s pre-treatment DT, and DT change across treatment, were significantly correlated with child OCD symptom improvement at post-treatment. The present study also extends these findings by suggesting a possible bi-directional association between DT change and OCD symptom change. DT is a family construct new to the child/adolescent OCD literature and warrants further attention and investigation.

Contrary to findings by Piacentini et al. (2011), cross-lagged panel models exploring associations between FA and child/clinician- rated OCD severity did not identify any significant predictors of change, despite showing good fit with the respective data. Notwithstanding the small sample size, one possibility is that FA and OCD symptoms may operate somewhat in parallel, suggested by meta-analytic findings by McGrath and Abbott (2019) that showed the number of family factors targeted in treatment significantly moderated FA, but not OCD, outcomes. Alternatively, there may be a dynamic and synergistic association between FA and OCD symptoms, where reductions in one variable facilitate reductions in the other in a bi-directional feedback loop of improvement. However, the current bivariate analyses were exploratory and the sample size was small, warranting replication with a larger sample.

Exploratory crossed-lagged panel models exploring associations between child-rated Blame and OCD symptom severity identified several predictors of change. Contrary to our expectations, parent- and child- rated OCD severity models both showed reduced OCD severity at Baseline significantly predicted reduced parental Blame at the subsequent treatment phase, Early Treatment. Similarly, in the clinician-rated model, a reduction in OCD severity at Mid Treatment significantly predicted lower levels of parental Blame at the subsequent treatment phase, Late Treatment. These findings may extend previous findings that parental Blame of the young person predicts OCD severity (e.g., Peris et al., 2012b) by highlighting a possible bi-directional association. Reduced Blame may occur during the process of OCD symptom change, or even as a result of this change. The association between Blame and OCD symptom severity would benefit from further investigation.

## Limitations and Future Directions

Notwithstanding notable strengths, including the use of temporal tracking methodology, the unique exploration of univariate patterns of change across treatment phases for a range of family factors, the use of multiple informants, and the unique exploration of bivariate associations between novel family factors and OCD symptom severity across treatment phases, this preliminary study would benefit from replication with a larger sample. A multi-site collaboration may be necessary in order to assemble a large sample of young people with OCD and their families for this purpose. Future research with a large pediatric dataset would also be a valuable extension of the current study with the ability to analyze session-by-session change over the course of treatment. A larger sample would allow for an RCT to be conducted, which could include additional conditions, comparing family-based CBT with standard CBT and a range of control conditions. Despite this study warranting replication, these early results are promising with similar patterns identified across informants for OCD symptom change and family factor change across treatment phases. Limited research has been conducted to date on family environment factors in child and adolescent OCD, consequently, measurement of these family constructs requires further attention. Some family factors, such as FA, are better assessed than some of the newer constructs in the child/adolescent OCD field, such as DT, Blame, Conflict, and Cohesion. As such, recommendations for future research include directing efforts at better understanding and assessing these newer family constructs. An additional consideration concerning measurement of parental behavior (e.g., FA, DT, Blame) is that parents are aware their behavior is being evaluated in relation to their child’s symptoms. It is possible that parental factors, such as social desirability or increased focus on the variables being assessed, could have impacted parental responses. Despite this possibility, parents still did report undesirable characteristics, such as Blame, and similar patterns emerged for respective variables across multiple informants. Future treatment studies would benefit from maintaining the current inclusion of multiple informants for assessment measures, including child-, parent-, and clinician- report, where possible. In addition, the inclusion of observational data, such as data from parent-child interaction tasks, would enrich our understanding of family constructs by providing behavioral information difficult to obtain from self-report measures. Moreover, clinical interviews with parents about family factors, such as blame and conflict, and the use of clinician-rated scales could assist in identifying any discrepancies between self-report baseline ratings and clinician ratings for these constructs. The current study identified bivariate patterns during preliminary analyses exploring associations between family factor change and OCD symptom change across treatment worthy of further investigation. Temporal tracking methodology, involving regular assessment periods over the course of treatment, is key to better understanding these bivariate associations. Tracking measures used in future studies need to be brief and accurately assess relevant constructs within a specific time frame (e.g., over the past week). Our investigation of change processes throughout best-practice OCD treatment for young people has provided a base for future research to explore associations between family factor change and OCD symptom change with the aim of improving functioning for young people with OCD and their families.
